# The role of quality improvement in radiography

**DOI:** 10.1002/jmrs.524

**Published:** 2021-07-02

**Authors:** Don J. Nocum, John Robinson, Warren Reed

**Affiliations:** ^1^ San Radiology & Nuclear Medicine Sydney Adventist Hospital Wahroonga New South Wales Australia; ^2^ Discipline of Medical Imaging Science, Sydney School of Health Sciences, Faculty of Medicine and Health The University of Sydney Sydney New South Wales Australia; ^3^ Medical Imaging Optimisation and Perception Group (MIOPeG), Discipline of Medical Imaging Science, Sydney School of Health Sciences, Faculty of Medicine and Health The University of Sydney Sydney New South Wales Australia

## Abstract

This editorial discusses the importance of quality improvement and quality assurance in the provision of medical imaging services, by exploring two studies which aim to improve the quality of practice in emergency departments (ED). The quality of work by ED radiographers are continually planned, measured, assessed, and improved to enhance patient care outcomes – from the accurate diagnosis of patients, maintaining the consistency of diagnostic images, and to minimising radiation exposure to patients.

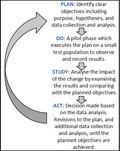

This issue of the *Journal of Medical Radiation Sciences (JMRS)* features two interesting articles which performed retrospective quality improvement studies on digital radiography in an Australian metropolitan emergency department (ED). The first study (Alexander‐Bates et al.)^1^ involved the investigation of a radiographer preliminary image evaluation (PIE) system, highlighting the most common false‐negative interpretations by cross‐correlation of the radiographer’s evaluation of any suspected pathology on x‐ray with the radiologist’s report. Their study used a clinical audit to assess the quality improvement on PIE accuracy within their ED and identified that most of the false‐negative radiographer PIEs were within upper and lower distal extremities. The second study (Stephenson‐Smith et al.)^2^ analysed the projection‐specific reject rates and radiographic examinations with multiple rejects and found that projections frequently repeated were horizontal beam lateral knee and horizontal beam hip. The authors addressed the use of reject analysis as a quality assurance strategy to minimise the need for repeat imaging.

It is useful at this point to compare and contrast the terms ‘quality assurance’ and ‘quality improvement’ in medical imaging. Quality assurance (QA) uses a systematic collection and evaluation of data to ensure the production of consistently high‐quality images with minimum exposure to patients and staff.^3^ Stephenson‐Smith et al.^2^ demonstrated that their reject analysis conforms to the QA model as the authors aimed to evaluate their overall reject and multiple reject rates. However, although useful, QA assumes that if problems or failures are inspected and eliminated, then what remains is considered of acceptable quality. This can embrace a philosophy that accepts quality as what is ‘good enough’, rather than what is the ‘best possible’ outcome. QA is also often considered judgemental and often perceived as punitive, eliciting potential fear, resentment and denial from practitioners.^4^ Quality improvement (QI) on the other hand is an umbrella term that includes (i) QA programs for monitoring quality improvement, (ii) processes to improve staff and patient safety and (iii) procedures to improve the clinical, technical and diagnostic performance of all staff.^4^ As addressed by the authors, projection‐specific reject and multiple reject analysis is important for QI to reduce patient radiation exposure.^2^ Alexander‐Bates’ et al.^1^ study is also recognised as a long‐term QA study due to their large monthly sample size (*n* = 100) deemed adequate for a local clinical audit according to the Royal College of Radiologists.^5^ Both studies examined two important aspects of medical imaging – PIE and reject analysis, which are critical for improving the quality of everyday services and patient care.

The *JMRS* regularly publishes clinical audits and retrospective studies that investigate aspects within the medical imaging department and its services that require improvement. There is an assumption that the audit will improve practice in the longer term, but this can only be demonstrated by follow‐up research using the advice to educate and monitor changes from the respective authors of these two studies.^1^, ^2^ Alexander‐Bates et al.^1^ and Stephenson et al.^2^ are both QA studies which focus on human error that seek to identify and reduce outliers or poor performance as a method. QA is an activity that is part of QI, which is required to establish an advanced confidence that performance is maintained at a high standard. Reducing false‐negative interpretations and multiple rejects are prime examples of striving to ensure that individuals are adhering to policies, procedures and protocols of the department that meets the standards required by regulatory and accrediting bodies.^4^

The next logical step is that each retrospective QA study or audit should be included as part of a continuous quality improvement cycle to actively monitor the impact of education and training on changes in practice and outcome over time. Continuous quality improvement (CQI), also known as total quality management (TQM), is a specific process that can be applied to medical imaging to further improve the quality of practice.^4^ CQI involves a cycle of identifying a practice within the medical imaging department that requires improvement, making observations and planning tests and analysing the effects of changes on the outcome and then asks the question, ‘what did we learn from this process?’ The entire cycle is repeated continuously to modify changes and delve into the possibility of other avenues for improvement.^6^ One of many CQI frameworks that are used in healthcare is known as the Plan‐Do‐Study‐Act (PDSA) cycle, or Deming cycle, which is described in Figure 1.^7^ Engineer and quality expert Dr W. Edwards Deming defined quality as being “on target, with minimum variance”.^7^

**Figure 1:**
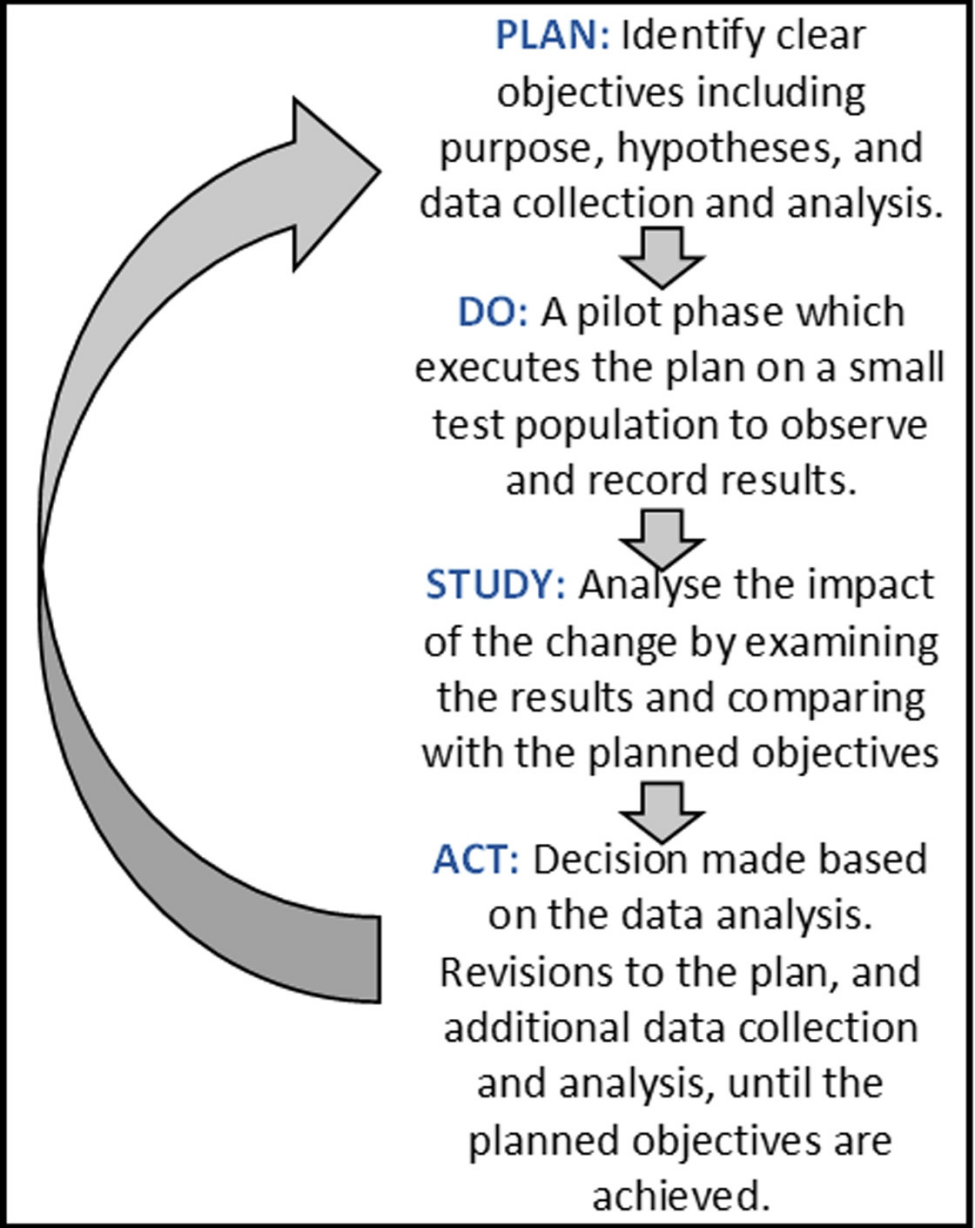
PDSA or Deming Cycle.^7^

CQI involves both prospective and retrospective reviews and is aimed at measuring where you are currently and then creating systems to make things better. It would be interesting to see the progression of longitudinal research for these two important studies^1^, ^2^ to assess the improvement on the measured gaps in the current system. To provide an example of the use of the PDSA cycle for a future study related to Alexander‐Bates et al.,^1^ a plan could be to improve image interpretations for all ED x‐rays, particularly for the ankle, foot, wrist, hand and phalanges. Educating and training radiographers about common false‐negative errors, correct pathology detection and ‘subsequent search miss (SSM)’ errors for multiple pathologies would assist these objectives. The cycle could then assess the performance of all radiographers and then analyse the PIE performance for interpreting pathology related to the upper and lower distal extremities to note any improvement in practice. Iterative data collection and evaluation would allow for assessing whether the objectives of educating and training radiographers had improved the quality of image interpretation. Monitoring for incremental and perpetual improvements in the quality of care can be achieved through continual review and reapplication of the PDSA cycle within the department. As this study suggests, ‘*education would allow radiographers to communicate “urgent and unexpected findings” to referrers, assisting in the treatment of patients and reducing missed pathology’*.^1^ This issue of JMRS also includes a study by Galli et al.^8^ which involved an image quality review programme for mammography based on the 2006 European guidelines for QA in breast cancer screening and diagnosis. Interestingly, this longitudinal study did follow a CQI model where training and monitoring of entry‐level and experienced radiographers developed the mammogram service and improved technical assessments of image quality.^8^ This is a key example of an effective QA study for continuous quality improvement for sustaining a high standard of diagnostic imaging and performance of radiographers.

A previous study,^9^ as part of an overall CQI program by the authors of this editorial, identified dose predictor variables for the interventional radiology procedure uterine artery embolisation (UAE). This has formed part of a QA process which has progressed to further studies which aim to fully implement this CQI program for optimising radiation dose during UAE by clinically validating the resultant regression model.^9^ The results have demonstrated significant dose reduction, which is critically important for these reproductive‐age UAE patients. This demonstrates that implementing CQI programs to improve quality of care can be beneficial to many medical imaging department procedures. Similar to the study by Stephenson‐Smith et al., the aim is to maintain ‘*a high standard of image quality whilst minimising radiation exposure to as low as reasonably achievable (ALARA)”* for the patient.^2^ Therefore, a follow‐up prospective study to the Stephenson‐Smith et al.’s^2^ study involving education of radiographic positioning and exposures of ED imaging (particularly of the pelvis, hip, spine and knee) would also be very useful to examine the possible improvements in the overall reject rate and multiple reject rate.

These two significant studies^1^, ^2^ included in this issue clearly signal the right direction for radiographers in using quantitative and qualitative research to improve the overall quality of our profession. Quality improvement in the ED is important as this is an area where we can be working to full scope of practice and provide accurate image interpretation to guide clinical and treatment decisions (i.e. PIE) and identify and correct errors in the system (i.e. reject analysis). Progressing this research from QA studies towards the concept of CQI will support radiographers in contributing to high‐quality, evidence‐based practice, leading to a positive impact at the level of the medical imaging department, the overall organisation and most importantly the patient.

## Conflict of Interest

The authors declare no conflict of interest.
